# Face mask mandates alter major determinants of adherence to protective health behaviours in Australia

**DOI:** 10.1098/rsos.241941

**Published:** 2025-03-26

**Authors:** Matthew Ryan, Jinjing Ye, Justin Sexton, Roslyn I. Hickson, Emily Brindal

**Affiliations:** ^1^ Commonwealth Scientific and Industrial Research Organisation (CSIRO), Adelaide, South Australia, Australia; ^2^ James Cook University, Townsville, Australia; ^3^ School of Civil Engineering, University of Sydney, Sydney, New South Wales, Australia

**Keywords:** face masks, policy mandates, machine learning

## Abstract

Face mask wearing is a protective health behaviour that helps mitigate the spread of infectious diseases such as influenza and COVID-19. Understanding predictors of face mask wearing can help refine public health messaging and policy in future pandemics. Government mandates influence face mask wearing, but how mandates change predictors of face mask wearing has not been explored. We investigate how mandates changed predictors of face mask wearing and general protective behaviours within Australia during the COVID-19 pandemic using cross-sectional survey data. We compared four machine learning models to predict face mask wearing and general protective behaviours before and after mandates started in Australia; ensemble, tree-based models (XGBoost and random forests) performed best. Other than state, common predictors before and after mandates included age, survey week, average number of contacts, wellbeing, and perception of illness threat. Predictors that only appeared in the top ten before mandates included trust in government, and employment status; and after mandates were willingness to isolate. These distinct predictors are possible targets for future public health messaging at different stages of a new pandemic.

## 1. Introduction

Effectively worn, high-quality face masks can be used as a low-cost protective health behaviour to mitigate the spread of infectious diseases such as influenza and COVID-19 [1,2]. Face masks were critical in the early stages of the COVID-19 pandemic in controlling the virus, with many models assessing the impacts and benefits to society [3–5]. Due to the potential of face masks to reduce infection transmission, understanding the social and behavioural drivers that predict face mask wearing may help increase public uptake in future pandemics through identifying key targets for public health messaging and policy. These features could also be used to inform and parameterize more realistic mathematical models of infection spread that accurately account for human behaviour, such as the recent model by Ryan *et al*. [6], which could better inform policy for future pandemics.

There are many existing identified drivers of face mask wearing, including socio-demographic [7–10], societal [7,10,11], cultural [12] and individual-level predictors [10,13–15]. However, face mask wearing is heavily influenced by enforced policy [9,16], which can increase mask usage by up to 90% [8]. Due to the high effect of policy on face mask wearing, understanding if key drivers of face mask wearing change before mandates are enacted compared to after periods of prolonged mandates start can help inform targeted health messaging. It has been shown that before mandates messaging may help increase voluntary adoption of face masks [17], whereas after mandated periods messaging may be used to help combat possible pandemic fatigue [7]. Furthermore, factors such as an individual’s altruism may be associated with voluntary health behaviours more so than mandatory ones [18,19].

The role of mandates in face mask wearing has been acknowledged and accounted for in previous analyses [7,9,10,13,14,16]. Petherick *et al*. [7] controlled for mandate policies while investigating the hypothesis of pandemic fatigue of protective health behaviours including face mask wearing and social distancing, finding that long-term adherence to high-cost protective behaviours such as social distancing is likely to wane over time, whereas low-cost behaviours like face mask wearing appear stable over time. Huang *et al*. [5] investigated how face mask mandates affected incidence rates of COVID-19 by controlling for a pre-mandate period in their analysis. They found that face mask mandates had a significant effect at reducing case numbers and death incidence from COVID-19. Finally, Binter *et al*. [13] found that empathy, germ aversion, and higher age were all related to increased wearing of face masks while controlling for whether masks had been mandated or not; interestingly, perceived susceptibility was not a significant predictor in their analysis. Despite acknowledging the possible role of mandates through accounting for them in statistical methods, no one has specifically focused on how the predictive features of health behaviour compliance change once a mandate period has been enacted and how these could extend beyond a single behaviour.

Protective behaviours rarely appear in isolation; for example, mask wearing is correlated with other protective health behaviours such as social distancing and hand hygiene [20]. Other authors have investigated the role of specific drivers in face mask wearing and general health behaviours, including collectivism [11], science belief [21], behavioural science constructs [12,14,22–24] and pandemic fatigue in continued adherence [7]. Largely exploratory analyses, rather than explanatory, have investigated key features that predict protective health behaviours, including empathy for others [9,13,25], perception of illness threat [10,13], trust in the government [13,25], political alignment [15,26] and social and cultural norms [9,10]. Schumpe *et al*. [10] provide a thorough exploration in a multi-country longitudinal cohort of different personal, social and country-level drivers of personal hygiene, face mask wearing and social distancing. The authors report that perceived risk was a common predictor of hand hygiene and social distancing, whereas proximity to actual COVID-19 cases was more predictive of face mask wearing. However, how these drivers change due to mandates being enacted has not been investigated.

Our aim was to investigate the common and distinct features that predict face mask wearing in Australia, before face mask mandates are enacted and after mandate periods start. Australia makes an interesting case study for such analyses, as the large geographical area and differing local government polices made for vastly different COVID-19 experiences across the states [27]. Given the likelihood that other protective health behaviours will be highly predictive of face mask wearing in any model we investigate, we also explored features that predict adherence to general health behaviours. The specific research questions were:

— What are the common and distinct features that predict face mask wearing in Australia before face mask mandates are enacted and after mandate periods start?
— What are the common and distinct features that predict general protective health behaviours (face mask wearing, social distancing, hand hygiene, etc.) in Australia before face mask mandates are enacted and after mandate periods start?

To explore these research questions, we accessed two freely available public good datasets: (i) the Imperial College London YouGov Behaviour Tracker dataset [28], which cross-sectionally tracked self-report survey responses around protective health behaviours in response to COVID-19 between 2020 and 2022 across 29 countries, and (ii) the Oxford COVID-19 Government Response Tracker dataset [29] which documented worldwide government responses and policies aimed at mitigating the spread of COVID-19. On these datasets, we aimed to use different machine learning models to identify the stable features that are commonly most predictive of adherence.

## 2. Methods

This study is a retrospective secondary analysis of cross-sectional survey data captured through YouGov and Imperial College London [28] combined with the Oxford COVID-19 Government Response Tracker [29]. This study was approved by the CSIRO Health and Medical Human Research Ethics Committee Low-Risk Review Panel (LR 009/2024).

We first describe the datasets used in this analysis and the data cleaning involved. We then describe the measures from the survey used to construct our response and features. Next, we describe the models investigated to predict face mask wearing and general health behaviour adherence. Finally, we describe how we evaluate model performance.

### 2.1. Data

#### 2.1.1. YouGov survey data

From 29 March 2020 to 3 March 2022, the Institute of Global Health Innovation (Imperial College London) and YouGov collected survey responses on human behaviour from 29 countries across the world, including Australia [28]. The survey was designed to collect nationally representative data on aspects of protective health behaviour in response to COVID-19. Data collected in the survey included demographic data (such as binary gender, employment, age, etc.), scaled items for protective health behaviours (such as mask wearing, social distancing, hand sanitizer usage, etc.), mental health and wellbeing, comorbidities and more. In Australia, a total of 513 items were recorded for 53833 participants over the course of the survey, with different items asked at different times throughout the survey; for example, questions relating to vaccine uptake and hesitancy were added later in the survey period.

Many items in the YouGov survey were asked sporadically or not at all in Australia, with responses to items asked ranging from 100% to less than 0.001%. Due to this, not all survey items can be used as features in our machine-learning models. We heuristically optimized the number of features included to minimize the number of observations lost due to missing data and to keep a signal of face mask wearing representative of the full dataset; the heuristic process is detailed in the electronic supplementary material. This resulted in 51 features for data from 24 June 2020 until 1 March 2022, removing 10781 observations. Specific measures used from the survey data are described in §2.2.

#### 2.1.2. Government mandates

Australia, like many places across the world, experienced varied government-enforced mandates between 2020 and 2022 due to the COVID-19 pandemic including face mask mandates, closed businesses and extended and snap lockdowns [27]. Due to Australia’s large geographical area and different local government responses between the states, each state had vastly different experiences with COVID-19. For example, Victoria had one of the longest continuous lockdowns in the world [30].

To account for the differing mandates in each state, we use the Oxford COVID-19 Government Response Tracker (OxCGRT) dataset [29], which provides readily usable information on policies across jurisdictions around the world, with state-level information for Australia. The OxCGRT dataset reports daily policy measures from March 2020 until January 2023 across Australian states covering containment and closure policies, economic policies, health system policies, vaccination policies and miscellaneous policies. We focus on the policies regarding facial coverings (column identifier H6) due to our interest in predicting face mask wearing during COVID-19 in Australia. The face mask policies are recorded as a five-point ordinal scale capturing no policy, recommendations only, requirement in specific public settings, requirements whenever social distancing is not possible, and requirements in all settings.

### 2.2. Measures

The final set of features in the model falls broadly into the five categories: (i) Self-protective behaviours; (ii) Demographics; (iii) Trust in government; (iv) Health, mental health and wellbeing; and (v) Perception of illness threat. We also included the survey period (measured in fortnights since the survey began) as a covariate in the models. The measures in each of these categories are described below, and a full list of included features is found in electronic supplementary material, table S1.

#### 2.2.1. Self-protective behaviours

Individuals were given 17 different self-protective behaviours in different scenarios to consider in response to the question ‘Thinking about the last 7 days how often have you taken the following measures to protect yourself or others from coronavirus (COVID-19)? As a reminder, please exclude any measures that you have already taken for reasons other than coronavirus (COVID-19)’. These scenarios may be categorized into face mask wearing (e.g. worn a face mask outside your home or in a supermarket), social avoidance (e.g. avoided going out in general or small/medium/large gatherings), hand hygiene (e.g. washed your hands with soap or used hand sanitizer) or coughing etiquette (e.g. covered our nose and mouth when sneezing or coughing). Responses to each of these scenarios were measured on a five-point scale ranging from ‘1 – not at all’ to ‘5 – always’.

Responses to these 17 scenarios were combined into three separate numeric variables, similar to Reitenbach *et al*. [31]. Face mask wearing was defined as the median response to the scenarios ‘Worn a face mask outside your home’, ‘Worn a face mask inside a grocery store/supermarket’, ‘Worn a face mask inside a clothing/footwear shop’ and ‘Worn a face mask on public transportation’. ‘Protective health behaviour adherence other than face masks’ was defined as the median response to all scenarios except for the four related to face mask wearing. General protective health behaviour adherence was defined as the median response to all scenarios.

Our two target variables, ‘Face mask wearing’ and ‘General protective behaviour compliance’, were defined as binary classes of their numeric counterparts. Face mask wearing/general protective behaviour was classified as 1 (wearing a face mask/general protective behaviour compliance) if their numeric value was 4 or above and 0 (not wearing a face mask/not general protective behaviour compliant) otherwise. ‘Protective health behaviours other than face masks’ was not converted to a binary variable and was used as a feature in the face mask prediction models only.

We also use three other questions on self-protective behaviours in our models. First is the average number of non-household contacts measured by ‘Not including those people in your household, about how many people have you come into physical contact with (within 2 metres/6 feet)?’ where individuals gave a single numeric value as a response. Willingness to isolate if unwell was measured by ‘Thinking about the next 7 days would you isolate yourself after feeling unwell or having any of the following new symptoms: a dry cough, fever, loss of sense of smell, loss of sense of taste, shortness of breath or difficulty breathing?’ where individuals could respond yes, no, or not sure. Finally, willingness to isolate if directed to was asked by ‘If you were advised to do so by a healthcare professional or public health authority to what extent are you willing or not to self-isolate for 7 days?’ with responses very unwilling, somewhat unwilling, neither willing nor unwilling, somewhat willing, very willing or not sure.

In these data, general protective behaviours were negatively associated with an average number of non-household contacts (Spearman correlation = −0.35) and positively associated with perception of illness threat (Spearman correlation = 0.30).

#### 2.2.2. Demographics

The demographic variables used in this analysis include age, gender (male/female), state, employment status and household size. The state was recorded as one of the eight Australian states or territories. Employment status was measured by asking ‘Which of these applies to you?’ with the options of full time employment, part-time employment, full-time student, retired, unemployed, not working or other. There were no responses in the ‘other’ or ‘full time student’ categories in the Australian data. Household size was measured with the question ‘How many people, including yourself, are there in your household? Please include both adults and children’, with the response options including sizes one to seven, eight or more, don’t know and prefer not to say. We converted household size to a numeric variable that is censored at eight people per household, that is, the n=190 people who responded with eight or more people per house were censored to eight per house. The n=767 people who responded with ‘don’t know’ or ‘prefer not to say’ were coded as missing and removed from the analysis. Household size and age had a moderate negative correlation in this dataset (Spearman correlation = −0.42).

#### 2.2.3. Trust in government

Trust in government was measured by two different questions. The first was ‘How well or badly do you think the Government are handling the issue of Coronavirus (COVID-19)?’ with the responses don’t know, very badly, somewhat badly, somewhat well and very well. The second was ‘How much confidence do you have in the Government to respond to a Coronavirus (COVID-19) outbreak in Australia?’^1^ with responses don’t know, no confidence at all, not very much confidence, a fair amount of confidence and a lot of confidence. The responses to these two questions were moderately correlated in this data set (Cramer’s V = 0.47), suggesting that these questions are partially exchangeable in the feature space.

#### 2.2.4. Health, mental health and wellbeing

Comorbidities were measured by asking ‘Which, if any, of the following have you been diagnosed with? Please select all that apply’, with a list of 13 options including, for example, cancer, chronic obstructive pulmonary disease and diabetes. Due to data sparsity for each comorbidity, we condensed this into a single categorical variable indicating whether they have a comorbidity, do not have one, or would prefer not to say.

Mental health was measured using the ultra brief Patient Health Questionnaire for Depression and Anxiety (PHQ-4) [32]. This scale asks ‘Over the last 2 weeks, how often have you been bothered by the following problems?’ to obtain response to (i) ‘Little interest or pleasure in doing things’, (ii) ‘Feeling down, depressed, or hopeless’, (iii) ‘Feeling nervous, anxious, or on edge’, and (iv) ‘Not being able to stop or control worrying’. Each of these questions had the response options not at all, several days, more than half the days, nearly every day and prefer not to say. Responses to each item in the PHQ-4 scale were correlated in this dataset (Cramer’s V = 0.66), suggesting any importance of these features may be exchangeable in the following predictive analysis.

Wellbeing was measured using the Cantril Ladder [33], which asks ‘Please imagine a ladder with steps numbered from zero at the bottom to 10 at the top. The top of the ladder represents the best possible life for you and the bottom of the ladder represents the worst possible life for you. On which step of the ladder would you say you personally feel you stand at this time?’. Responses to this item ranged from zero (low wellbeing) to ten (high wellbeing) and were treated as a numeric value.

Due to how consent was asked in the survey between 19 February 2021 and 18 October 2021, many records for health-related questions (PHQ scale and comorbidities) were removed from the dataset and coded as a categorical ‘NA’. The categorical NA’s introduced to the data also introduce a correlation between the presence of comorbidities and responses to the PHQ scale (Cramer’s V = 0.56).

#### 2.2.5. Perception of illness threat

The perception of illness threat was measured using two questions. Perceived susceptibility was measured with the question ‘To what extent do you agree or disagree that coronavirus (COVID-19) is very dangerous for me?’ while perceived severity was measured with ‘To what extent do you agree or disagree that it is likely I will get coronavirus (COVID-19) in the future?’ Both questions were measured on a seven-point scale ranging from ‘1 – Disagree’ to ‘7 – Agree’ which were converted to a numeric value for analysis. Responses to these two questions were mildly correlated in this dataset (Spearman correlation = 0.30).

### 2.3. Data splitting

The data were split into two subsets, before mandate data and after mandate data, based on the OxCGRT dataset. Many states had periods of minor mask mandates sporadically from March 2020, but most states had continuous mandates from approximately the start of July 2021. To separate our data into periods before mandates fully came into effect, and periods of prolonged mandates, we define the ‘before mandate’ period to be all data in a state before continuous mandates were enacted and define the ‘after mandate’ period as any time after this. That is, a binary time-split of the data before the continuous mandates or after they were first enacted. We define a period of continuous mandates as a period of at least 14 days with face masks required whenever social distancing is not possible, similar to White *et al*. [14]. Given the facial covering policies were scaled between 0 (no policy) and 4 (required in all settings), and at times policies changed over short time periods, we defined the average policy over a 14 day period as the rolling average of the policy index for the preceding 14 days. Based on the average policy, continuous mandates were defined to be in place when this value was 3 or above, selected for both compliance with the White *et al*. [14] aligned definition, and after inspecting the rolling averages (see electronic supplementary material, figure S1). Using this definition, continuous mandates first came into place for Victoria in July 2020, and last came into place for the Northern Territory in November 2021. A notable limitation of this approach is the vastly different experience with COVID-19 that each state within Australia experienced. In particular, face mask mandates started in Victoria on 23 July 2020, which means less than a month worth of data for Victoria are available in the before-mandate dataset.

All missing values were removed resulting in 40136 (before mandates =14945, after mandates =25191) observations in the final dataset. Each dataset was split into training (80%; before mandates =11956, after mandates =20152) and validation (20%; before mandates =2989, after mandates =5039) sets to tune and validate the predictive models.

### 2.4. Analyses

We investigated four separate machine learning models to predict face mask wearing and general protective health behaviour. These models were chosen due to their readily available feature importance measures and include two baseline models (logistic regression and classification trees) and two more advanced models (XGBoost and random forests), described below. In the before mandate period, face mask wearing was heavily underrepresented in the data (26% wearing face masks; electronic supplementary material, table S1), whereas this relationship swapped after mandated periods (71% wearing face masks; electronic supplementary material, table S1). Thus, for each target variable (face mask wearing and general protective health behaviour) and each time period (before and after mandates), we randomly up-sample the minority class within the training set, to have balanced datasets for classification.

All models were fitted using scikit-learn 1.4.2 and xgboost 2.0.3 in Python 3.12.3. All plots were made in R 4.3.1. The Python code was run on a high-performance computing Dell PowerEdge C6525 Linux cluster. All code is available at https://github.com/Matthew-Ryan1995/face_mask_predictors.

#### 2.4.1. Logistic regression

Logistic regression is a statistical model that predicts the probability of being in a class given a set of feature variables. It constructs a linear classifier on the logit scale and has previously been applied to face mask wearing [13,34]. Feature importance for logistic regression is taken as the beta coefficients of the model, with large positive values indicating that the feature is predictive of the target class (for example, wearing a face mask) and large negative values indicating that the feature is predictive of the negative class (for example, not wearing a face mask).

#### 2.4.2. Classification trees

Classification trees are a type of decision tree that constructs a series of yes/no questions that classify observations into the positive or negative class [35]. As such, classification trees are fast and highly interpretable. Feature importance in decision trees is measured by how much the predictive accuracy of the model is reduced by removing individual variables, hence features with a high relative importance lend more to the predictive accuracy of the model.

#### 2.4.3. XGBoost

XGBoost (extreme gradient boosting trees) is an ensemble method that sequentially builds decision trees, using information from the previous tree to improve the predictions of the subsequent trees, known as boosting [36]. Ensemble methods work by training a lot of models and averaging the results to get a single prediction— for classification problems, this averaging amounts to taking the most popular class predicted by each model in the ensemble. XGBoost is a greedy algorithm that leverages key features identified in previous trees, making these features more likely to appear in subsequent trees. Similar to classification trees, feature importance is measured by how much predictive accuracy is reduced when a particular feature is removed from the model.

#### 2.4.4. Random forests

Random forests are another ensemble tree-based method like XGBoost, but with some distinct difference [37]. Firstly, rather than boosting new trees based on the performance of the older trees, random forests employ bagging [38]. Bagging bootstraps the training data—that is, randomly samples from the training data with replacement—to build new datasets to train decision trees on, creating a ‘forest’ of decision trees. The other key difference is that random forest randomly sample the number of features to consider every time a decision is made in each tree of the forests. This ensures that the trees in the forest are not correlated in their predictions, reducing bias in the predictions. Feature importance is calculated similarly to classification trees and XGBoost.

### 2.5. Model performance

#### 2.5.1. Model selection

For each outcome variable (face mask wearing or general protective behaviour) and each time period (before and after mandate periods), we tuned the hyperparameters of the tree-based machine learning models using the Python package optuna [39]; logistic regression was not tuned because it does not have any inherent hyperparameters. The optuna package explores the hyperparameter space of each model using a tree-structured Parzen estimator [40], which is a sequential global tree-based optimization algorithm to find optimal parameter combinations. Tuning was performed using fivefold cross-validation [41] on the training sets, optimizing the area under the receiver operator curve (AUC) [42]; see electronic supplementary material for a description of cross-validation.

An initial exploration of 250 hyperparameter combinations was explored to identify which hyperparameters were most influential on the AUC for each of the four models (target variable and mandate period). Once the most influential hyperparameters were identified, these parameters were tuned across 1000 parameter combinations. For both XGBoost and random forests, we fixed the number of estimators (trees) at 250 for the final tuning as this stabilized the AUC in the small-scale explorations.

The final models were chosen as the most parsimonious models within one standard error of the optimal AUC values found in the tuning process. For each model, parsimony was determined using the following steps:

— Find all tuples of hyperparameters that produce an AUC within one standard error of the optimal AUC value.
— Order the hyperparameter tuples by the most influential hyperparameter first, then the second most, and so on.
— The most parsimonious model is the model determined by the smallest hyperparameter tuple determined by the previous ordering.

#### 2.5.2. Evaluation metrics

The optimal models were chosen to maximize the AUC metric. The AUC measures how well the model predicts both positive and negative cases overall; for example, how well the model predicts when an individual is wearing a face mask and when they are not. In addition to AUC, we also recorded the precision, recall, accuracy, and F1 scores for each model. Precision captures the proportion of times that predicted positive cases from the model are true positive values. For example, when our model predicts an individual as wearing a face mask, how often is it correct? Recall is a similar metric, but measures the proportion of times that positive cases are captured by the model. That is, how often do we expect our model to correctly predict an individual as wearing a face mask. Accuracy captures the proportion of time our model correctly predicts the data, both positive and negative cases. F1 is an average of precision and recall, capturing a trade-off between the two. A nice mathematical description of each metric can be found in [43].

#### 2.5.3. Sensitivity on feature importance

Once the optimal models had been chosen through cross-validation, we investigated the important features in the models. However, randomly up-sampling the minority class in each model introduces potential randomness into the model predictions and feature importance Thus, we investigated the stable features important to each model. To do this, for each target variable (face mask wearing and general protective health behaviour) and each time period (before and after mandates) we fitted the best machine learning models 10000 times and extracted the feature importance for each feature in the model. The ten most important stable features were identified as those with the highest median importance scores.

## 3. Results

### 3.1. Model performance and selection

Initial explorations of the hyperparameter spaces reduced the hyperparameters for each model down to at most four to be tuned. For classification trees, we tuned the minimum impurity decrease for a split to occur, the minimum weighted fraction for a node to be a leaf, the minimum samples to create a split or have a leaf node, and the maximum depth of the tree. For the XGBoost models, we tuned the boosting learning rate, the subsample ratio of the training instance to prevent overfitting, the subsample ratio of columns used to train each tree, the minimum weighting of samples to create a child node, the minimum loss required to make a split and the maximum depth of each tree. For random forests, we tuned the number of features to sample when creating a split, the minimum samples needed to make a split, the minimum samples needed to make a leaf node, and the maximum depth of each tree.

In cross-validation, both the random forest and the XGBoost models perform equivalently and optimally (highest AUC) in predicting face mask wearing and compliance with general health behaviours in before mandate periods, and XGBoost performs optimally in after mandate periods (table 1). Before mandate periods, the random forests have higher precision than XGBoost when predicting face mask wearing (0.657 vs. 0.549) and adherence to health behaviours in general (0.595 vs. 0.522). However, the XGBoost models have better recall compared to random forests when predicting face masks (0.754 vs. 0.576) and general health behaviours (0.725 vs. 0.584) in the same period.

**Table 1.** Fivefold cross-validation results comparing four predictive models for predicting face mask wearing and general protective health behaviours before and after face mask mandates are enacted. Values are given as mean (standard error) and can range from 0 (low) to 1 (high). **Bold values show the optimal models based on largest AUC**.

	AUC	precision	recall	accuracy	F1
before mandates—face mask wearing					
logistic regression	0.824 (0.004)	0.479 (0.003)	0.769 (0.011)	0.727 (0.002)	0.590 (0.005)
classification tree	0.811 (0.004)	0.486 (0.005)	0.753 (0.012)	0.733 (0.004)	0.591 (0.003)
XGBoost	**0.861 (0.001)**	0.549 (0.004)	0.754 (0.006)	0.778 (0.002)	0.635 (0.004)
random forest	**0.858 (0.002)**	0.657 (0.007)	0.576 (0.007)	0.814 (0.003)	0.614 (0.006)
after mandates—face mask wearing					
logistic regression	0.837 (0.002)	0.876 (0.001)	0.783 (0.002)	0.770 (0.001)	0.827 (0.001)
classification tree	0.866 (0.003)	0.892 (0.002)	0.840 (0.007)	0.816 (0.004)	0.865 (0.004)
XGBoost	**0.896 (0.002)**	0.895 (0.002)	0.891 (0.002)	0.850 (0.003)	0.893 (0.002)
random forest	0.888 (0.002)	0.890 (0.002)	0.890 (0.002)	0.845 (0.002)	0.890 (0.002)
before mandates—general protective behaviour					
logistic regression	0.777 (0.005)	0.438 (0.006)	0.734 (0.008)	0.691 (0.005)	0.549 (0.006)
classification tree	0.779 (0.004)	0.451 (0.005)	0.737 (0.011)	0.704 (0.004)	0.560 (0.007)
XGBoost	**0.834 (0.002)**	0.522 (0.005)	0.725 (0.007)	0.760 (0.004)	0.607 (0.005)
random forest	**0.829 (0.004)**	0.595 (0.007)	0.584 (0.005)	0.792 (0.003)	0.590 (0.005)
after mandates—general protective behaviour					
logistic regression	0.780 (0.003)	0.859 (0.003)	0.736 (0.002)	0.722 (0.002)	0.793 (0.002)
classification tree	0.798 (0.002)	0.879 (0.002)	0.690 (0.007)	0.708 (0.005)	0.773 (0.005)
XGBoost	**0.845 (0.002)**	0.885 (0.002)	0.783 (0.002)	0.770 (0.001)	0.831 (0.001)
random forest	0.836 (0.002)	0.854 (0.001)	0.853 (0.002)	0.789 (0.001)	0.854 (0.001)

After mandates are enacted, the same precision and recall trade-off is observable between the random forest and XGBoost when predicting general protective health behaviours, but both models perform equivalently on all measured model metrics (except AUC) when predicting face mask wearing.

Since the cross-validation results for the random forest and XGBoost models were equivalent in the before mandate periods, we fitted both models to the training data using the optimal tuning parameters to investigate key predictive features in the models. This resulted in eight final models; a random forest and an XGBoost model each for face masks and health behaviour prediction in before and after mandate periods. The final models were fitted using a single seed for the upsampling of the minority class in each situation. The performance metrics on the validation dataset are consistent with, but slightly worse than, the cross-validation results (table 2); note that XGBoost marginally outperforms the random forest in AUC across all models. Further, observe that the before-mandate models show the same trend in precision and recall as seen in the cross-validation.

**Table 2.** Metric evaluation on an independent validation set for the optimal models predicting face mask wearing and general protective health behaviours before and after face mask mandates are enacted. Metric scores can range from 0 (low) to 1 (high).

	AUC	precision	recall	accuracy	F1
before mandates—face mask wearing					
XGBoost	0.853	0.561	0.746	0.776	0.640
random forest	0.848	0.666	0.576	0.809	0.618
after mandates—face mask wearing					
XGBoost	0.888	0.901	0.884	0.846	0.892
random forest	0.880	0.891	0.890	0.842	0.891
before mandates—general protective behaviour					
XGBoost	0.821	0.532	0.715	0.755	0.610
random forest	0.817	0.613	0.591	0.791	0.602
after mandates—general protective behaviour					
XGBoost	0.847	0.897	0.776	0.771	0.832
random forest	0.840	0.868	0.856	0.799	0.862

### 3.2. Comparison of key predictors before and after mandate periods

The dummy variables for the eight Australian states and territories were highly influential for predicting both face mask wearing and general health behaviour adherence, comprising between one and six of the 10 most consistent predictive features across each of the eight models. Further, every state or territory except for the Northern Territory appeared at least once in the top 10 most predictive features across the eight models. This is unsurprising given the different policies and COVID-19 experiences of each state within Australia [27]. As such, although states were included in the analyses to acknowledge their importance, we show the ten most consistently predictive features for each model other than state to investigate the other features that influence behaviour adherences (figures 1 and 2). Partial dependency plots for the identified features of interest were generated using the dalex package [44] in Python and are available in the electronic supplementary material (figures S12–S23 and S31–42).

**Figure 1.**
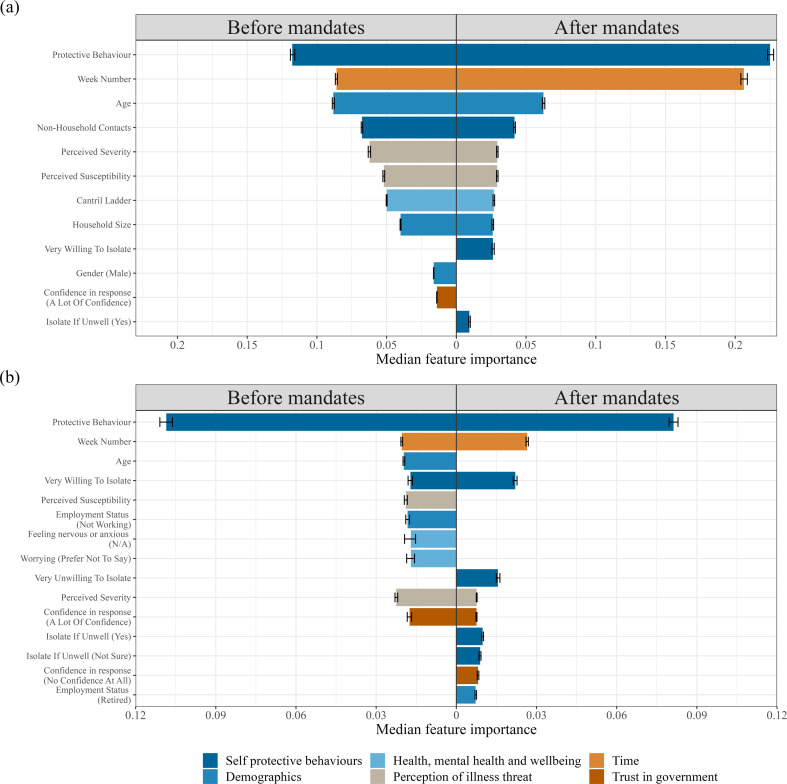
The top ten most influential features when predicting face mask wearing, other than state, using (a) a random forest and (b) XGBoost. Feature importance was ranked as the median importance value from 10000 fits of the model where randomness is induced by randomly upsampling the minority class for each fit. The error bars show the interquartile importance range for each feature. The left hand panel in each plot shows the feature importance before face mask mandates started and the right hand panel shows the feature importance after face mask mandates start. The bars are coloured based on the category of the feature as defined in the legend.

**Figure 2.**
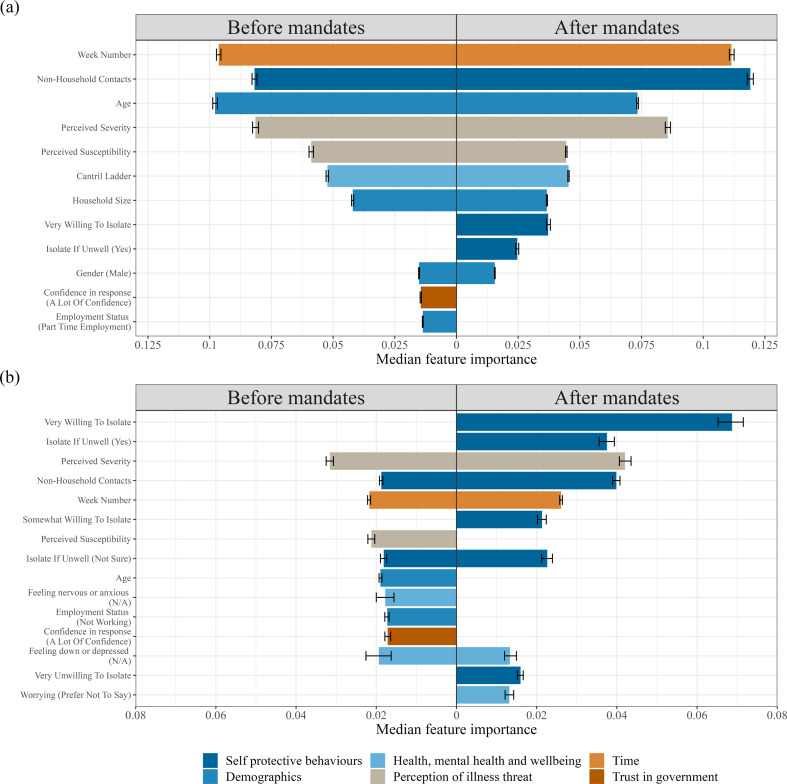
The top ten most influential features when predicting general protective health behaviours, other than state, using (a) a random forest and (b) XGBoost. Feature importance was ranked as the median importance value from 10000 fits of the model where randomness is induced by randomly upsampling the minority class for each fit. The error bars show the interquartile importance range for each feature. The left hand panel in each plot shows the feature importance before face mask mandates started and the right hand panel shows the feature importance after face mask mandates start. The bars are coloured based on the category of the feature as defined in the legend.

Both the random forest (figure 1a) and the XGBoost (figure 1b) identify other protective health behaviours and the time period of the survey (week number) to be the most influential predictors of face mask wearing, regardless of mandate period and typically increase compliance (electronic supplementary material, figures S12 and S14). The random forest suggests that several features are predictive of face mark usage both before and after mandate periods, specifically age, average number of non-household contacts, perception of illness threat, wellbeing and household size. Increased age and non-household contacts typically decrease compliance whereas increased perception of illness threat, wellbeing, and household size typically increase compliance (electronic supplementary material, figures S12 and S13). Distinct features that predict face mask wearing before mandates are gender (male) and confidence in the government’s response to COVID-19, and after mandates is willingness to isolate. The relationships between these features and mask usage depend on the response given to each question (electronic supplementary material, figures S12, S18 and S19).

The XGBoost model identified many distinct features of face mask wearing between the two periods. Unlike the random forest model, the XGBoost model suggests that age and perceived susceptibility to COVID-19 are only influential in face mask wearing before mandates are enacted. Other key features identified in predicting face mask usage before mandates include measures of individuals’ mental health, which are notably absent from predicting face mask wearing after mandates. Similar to the random forest, willingness to isolate or not are key predictive features of mask usage after mandated periods. Partial dependency plots of these relationships are consistent with the random forest models and available in electronic supplementary material (figures S15–S17 and S21–S23).

When predicting adherence to health behaviours in general, the random forest identifies many common features both before and after mandates (figure 2a). These features include the survey period (week number), the average number of contacts, age, perception of illness threat, wellbeing and household size. As with face mask usage, increased age and average number of contacts decreases compliance whereas the rest are positively associated with compliance (electronic supplementary material, figures S31–S33). Before mandates, distinct features of health behaviour compliance include employment status and confidence in the government’s response to the pandemic, whereas after mandate periods distinct features relate to willingness to isolate when instructed or if feeling unwell. As before, the relationship between compliance and these questions depends on the individuals’ response (electronic supplementary material, figures S31 and S38).

The XGBoost model suggests that perceived severity of COVID-19, average number of contacts and the time since the pandemic started (survey week) are all key predictors of protective health behaviour both before and after mandate periods. Before mandates, age and perceived susceptibility to COVID-19 are distinct features that predict health behaviour, unlike in the random forest where these features are predictive in both periods. Another notable feature to predict health behaviour before mandates is confidence in the government’s response to the COVID-19 pandemic. Similar to the random forest model, the key distinct predictors of health behaviours after mandates is someone’s willingness or unwillingness to isolate when instructed or unwell. Partial dependency plots of these relationships are consistent with the random forest models and available in the electronic supplementary material (figures S34–S36 and S40–S42).

## 4. Discussion

We have investigated the distinct and common features that predict face mask wearing and adherence to more general health behaviours in Australia before face mask mandates came into effect and after mandates started. We compared four common machine learning models with feature importance measures and found that random forests and XGBoost models were the most effective at predicting health behaviours, suggesting a nonlinear relationship between the feature set and health behaviours. Many common features were identified across both models that predict face mask wearing and general health behaviours before and after mandate periods. Notably, key common features were age, time since the beginning of the pandemic (survey week), average number of contacts, wellbeing and perception of illness threat. We also note that compliance with protective health behaviours other than face mask wearing is also a key feature in predicting face mask wearing during both periods. Distinct features, which only appeared in the top ten before mandated periods, predicting face mask wearing and health behaviours included trust in the government and employment status, whereas willingness to isolate only appeared in the top ten features after mandated periods.

Despite variations between the random forest and XGBoost models, there are some consistent themes. First, many of the key features identified here have been observed by others, including perception of illness threat [10,12–14,16], trust in government or science [10], age and other demographics [7–9,15] and a time effect [7]. Second, several of the key predictors can be aligned with the Health Belief Model [45,46], specifically perceived susceptibility and severity. Other variables may also be associated with the perception of illness threat, such as age and number of contacts, which were clearly identified risk factors for negative outcomes throughout the pandemic. These perceptual variables had stronger association than the presence or absence of chronic conditions. The Health Belief Model was designed specifically to predict optimal changes in health behaviours, which has been the focus of the current modelling, and our findings generally support this theory. Interestingly, newer iterations of the Health Belief Model have also included cues to action, which in the case of mandates and a visible health behaviour are salient. The Health Belief Model may be a useful model for identifying features that predict protective health behaviours, and has been successful in understanding face mask wearing during COVID-19 [14]. Further, the Health Belief Model has recently been embedded within a mathematical model for infection spread [6], so the present findings have the potential to inform further mathematical modelling. However, the available data used here does not include efficacy or cognitive evaluations of action (cost versus benefit). The measures included also failed to capture other key components of popular health behaviour models such as subjective norms or attitudes. Therefore, this analysis is by no means a validation of the Health Belief Model or support this over any other model.

Confidence in government was a significant feature in the before mandate period. Trust is central to the uptake of vaccines [47] but is likely to become less relevant as mandates ensue, at which time more cultural and individual factors are likely to predict compliance. In the absence of any more specific individual dispositional assessments, it is possible that willingness to follow direction of health professionals captures a level of dispositional compliance. Willingness to follow instructions can be captured by personality facets such as agreeableness and conscientiousness. Longitudinal data have supported the idea that behavioural compliance is higher in those with more agreeable personality traits [48]. Interestingly, agreeableness is also associated with compassion and empathy, which are also noted among the strongest individual predictors of protective health behaviours [49].

Wellbeing and mental health appear in some models, but are not consistently represented as key features. Coping self-efficacy [50] is a key concept from the wellbeing literature that may warrant future investigation. It is associated with wellbeing and has been identified as critical in how people are able to mitigate possible distress associated with traumatic large-scale events [51]. Unlike dispositional factors that may be more challenging to shift, coping self-efficacy can be improved through targeted interventions.

It has been observed that wider preventative health behaviours tend to cluster [52,53] and these analyses support this in the context of specific behaviours. Of relevance for public health officials is whether mandated behaviours can also promote other health protective actions, or whether the lack of autonomy associated with mandates interferes with possible upward spiralling. Available data limited our analysis to specific health actions that were captured consistently. Vaccine uptake was not among these and we are unable to reflect on any possible relationship between this and other health protective behaviours.

A limiting feature of our modelling is the strong effect that state has on predicting compliance with protective behaviours, which has been observed in the United States [8,15] as well as across countries [7,10,11]. A potential reason for this strong relationship with state is that mandates dictate the behavioural expectations, but the way in which they are enforced and implemented can also vary by context. For example, some states introduced significant fines [54] or targeted police enforcement operations [55]. Furthermore, although rarely considered in most analyses, there may be cultural, political and attitudinal differences between populations (for example, climate attitudes [56]). This is further exacerbated in our modelling because of the large geographical area of the states involved, where most observations likely come from the capital cities and the culture of regional areas is likely underrepresented. Future work may consider controlling for state as a random effect, which has found success at country-level analyses [16].

Although our results are inline with previously identified features related to protective health behaviours, they are limited by the data we have used. First, the Imperial College London YouGov dataset [28] is a versatile dataset that tracks many different health behaviours over time in many different countries. However, not all desirable features have been measured in these data, particularly over the full study period in Australia. In particular, there are no measurements of social or cultural norms that dictate behaviour, or any measurements around personality traits. Second, we have applied a binary view of mandates within Australia to the OxCGRT data. Although this simplifies our analysis and is somewhat representative of the study period, it does not capture the depth of complexity of the different policies that were implemented over the course of the COVID-19 pandemic. Further, we have only investigated the effects of face mask policies. It is likely the breadth of polices and the strength of policies all influenced health behaviour compliance over the course of the COVID-19 pandemic, so future work should consider a more complete approach to account for these policies.

There are many avenues to extend this work. Our modelling has focused on Australia, where each state had vastly different experiences with COVID-19. However, every country had different reactions, policies and cultural responses to the pandemic as well. A clear avenue to further this work is to extend this investigation to different countries around the world and explore the impact of different cultures on key predictors of health policy adherence. An obvious list of target countries are the countries included in both the OxCGRT and YouGov datasets. A key point to address in this future research is identifying a set of common and comparable features across the countries to inform the modelling. Another area important in guiding future pandemic response in Australia is to disentangle the state effect in predicting health behaviour adherence. In Australia, a lot of the COVID-19 response was guided by state-level governments, so understanding the differences in adherence across states is important to inform pandemic planning. However, most of the data available are from Victoria and New South Wales, which prohibits sufficient state-level analysis from the data used here. Investigating more wide scale and state representative data within Australia may help untangle the state-level differences, and is an area of future research. Finally, the data considered here does not include any measures of personal beliefs, political leanings or cultural backgrounds, all of which have been linked to health behaviour adherence [11,13,14]. One approach to address this is to combine the data used here with state-level data capturing social variables across different demographics (for example, the Australian census data [57]), which has been done in similar contexts [7,11]. However, this will prevent investigation of individual level drivers of health behaviour adherence.

By understanding the individual-level features that drive self-protective actions during a pandemic, we are in a better position to inform targeted policy and public health behaviour interventions. In doing this, it is key to recognize that these features may differ in the early stages of a new infectious disease compared to during extended periods of mandates and uncertainty. Leveraging the common and distinct features of health behaviour adherence can be used to develop and validate robust retrospective models that account for human reactions in different situations to explore optimal policy strategies, ultimately putting us in a better position to respond to future pandemics.

## Data Availability

The Imperial College London and YouGov behavioural data are available from their GitHub page [28]. The Ox CGRTpolicydata [29] are available from their GitHub page [58]. Data and relevant code for this research work are stored in GitHub: https://github.com/Matthew-Ryan1995/face_mask_predictors and have been archived within the Zenodo repository: [59]. Supplementary material is available online [60].

## Footnotes

^1^
Exact wording for Australian version of survey not available.
